# Evaluation of the effect of therapeutic durations on small ruminant bacterial pneumonia

**DOI:** 10.1186/s12917-024-03917-z

**Published:** 2024-02-24

**Authors:** Sisay Girma, Tesfaye Bekele, Samson Leta, Desiye Tesfaye Tegegne, Tilaye Demissie, Birhanu Hadush, Kassaye Aragaw, Takele Beyene Tufa, Teshale Sori Tolera, Ketema Tafess

**Affiliations:** 1 College of Veterinary Medicine and Agriculture, Addis Ababa University, Bishoftu, Ethiopia; 2 Institute for Microbiology, University of Veterinary Medicine Hannover, Hannover, Germany; 3 School of Veterinary Medicine, Wolaita Sodo University, Wolaita Sodo, Ethiopia; 4 National Agricultural Biotechnology Research Center, Ethiopian Institute of Agricultural Research Center, P.O. Box 249, Holeta, Ethiopia; 5 Department of Veterinary Clinical Medicine and Epidemiology, College of Veterinary Sciences, Mekelle University, Mekelle, Tigray Ethiopia; 6 Faculty of Veterinary Medicine, Hawassa University, P.O. Box 05, Hawassa, Ethiopia; 7 Institute of Pharmaceutical Sciences, Adama Science and Technology University, P.O. Box 1888, Adama, Ethiopia; 8 Department of Applied Biology, School of Applied Natural Science, Adama Science and Technology University, P.O. Box 1888, Adama, Ethiopia

**Keywords:** Sheep, Goat, Oxytetracycline, Respiratory diseases, Therapeutic duration, Ethiopia

## Abstract

**Background:**

Sheep and goat production in Ethiopia is hindered by numerous substandard production systems and various diseases. Respiratory disease complexes (RDC) pose a significant threat to the productivity of these animals. Pneumonia is a common manifestation of respiratory disease complexes and often necessitates a prolonged course of antibiotic treatment. This study aimed to optimize and propose the ideal duration of therapy for pneumonia in sheep and goats.

**Methods:**

The study was conducted from February to June 2021 at the Veterinary Teaching Hospital of the College of Veterinary Medicine and Agriculture, Addis Ababa University. The study recruited 54 sheep and goats presented to the hospital for treatment with a confirmed RDC as determined based on clinical signs and bacteriological methods. The animals were randomly allocated to 5 groups each group receiving 10% oxytetracycline (Phenxyl, Phenix, Belgum) intramuscularly for a duration of 3, 4, 5, 6 and 7 consecutive days. The treatment outcomes were assessed by recording vital signs (body temperature, respiratory rate, heart rate, coughing, and nasal discharges), performing lung ultrasonography (L-USG) as well as collection of nasal swabs for bacterial isolation and molecular identification before and after completion of the treatment. An ordered logistic regression model with random effects was employed to determine the optimal therapeutic duration, taking into account the cumulative scores of the outcome variables across the different groups.

**Results:**

Among the 54 sheep and goats treated with 10% oxytetracycline, a total of 74.07% (95% CI, 60.35–85.04) achieved complete recovery, as confirmed through clinical, ultrasound, and bacteriological methods. In Group 1 (G1), out of 12 sheep and goats, 8 (83.0%) recovered completely; in Group 2 (G2), out of 11 animals, 9 (82.0%) recovered completely; in Group 3 (G3), out of 11 animals, 10 (93.0%) recovered completely; in Group 4 (G4), out of 9 animals, 9 (100.0%) recovered completely; and in Group 5 (G5), out of 11 animals, 10 (91.0%) recovered completely. Bacteriological examination of nasal swabs indicated involvement of *M. hemolytica* in 27 (50.00%) and *P. multocida* in 13 (24.07%) of pneumonic animals. Detection of specific marker genes confirmed only five of the presumptive *M. hemolytica* isolates, whilst no isolates tested positive for *P. multocida*. Post-treatment samples collected from recovered animals did not yield any *M. hemolytica* nor *P. multocida*. Based on results from clinical signs, L-USG, and bacterial infection variables, the group of sheep and goats treated for seven consecutive days (G5) showed the highest recovery score compared to the other groups, and there was a statistically significant difference (coefficient (β) = − 2.296, *p* = 0.021) in variable score between G5 and G1. These findings suggest that the administration of 10% oxytetracycline for a full course of seven consecutive days resulted in symptomatic and clinical recovery rates from respiratory disease in sheep and goats.

**Supplementary Information:**

The online version contains supplementary material available at 10.1186/s12917-024-03917-z.

## Introduction

Respiratory diseases are among the serious threats to the sheep and goat industry in Ethiopia, especially in highland parts of the country, causing 54% of overall mortality in sheep [1]. It commonly occurs in the form of pneumonia under both intensive farms and extensive systems [2]. The common form of respiratory disease in sheep and goats is pneumonic Pasteurellosis, which is mainly caused by *Mannheimia hemolytica* and *Pasteurella multocida* [3, 4]. Since these bacteria are commensals in the upper respiratory tract, triggering factors such as environmental conditions, concurrent infections and poor nutrition predispose animals result in acute, chronic, and/or progressive pneumonia [5].

Antimicrobial therapy has been the basis of treatment for respiratory diseases for many decades. Drugs such as oxytetracyclines supplemented with nonsteroidal anti-inflammatory drugs (NSAIDs) are the recommended therapeutic arsenal [6–8]. In Ethiopia, it is common to observe antimicrobial treatment courses of 3 days, although most studies recommend a longer duration [9]. Empirical evidence has shown that treated animals suffer from recurrent pneumonia. Field veterinarians/para-veterinarians and livestock owners often raise complaints on the effectiveness of antimicrobial therapy. The results of in vitro sensitivity tests, however, showed that *M. hemolytica* and *P. multocida* are susceptible to commonly used antimicrobial drugs [10, 11]. We suggest that the short duration of therapy resulted in incomplete recovery and subsequent relapse of pneumonia in treated sheep and goats. This study was, therefore, aimed at investigating the optimum duration of treatment of pneumonia in sheep and goats using oxytetracycline (10%).

## Materials and methods

### Description of the experimental setup

**Fig. 1:**
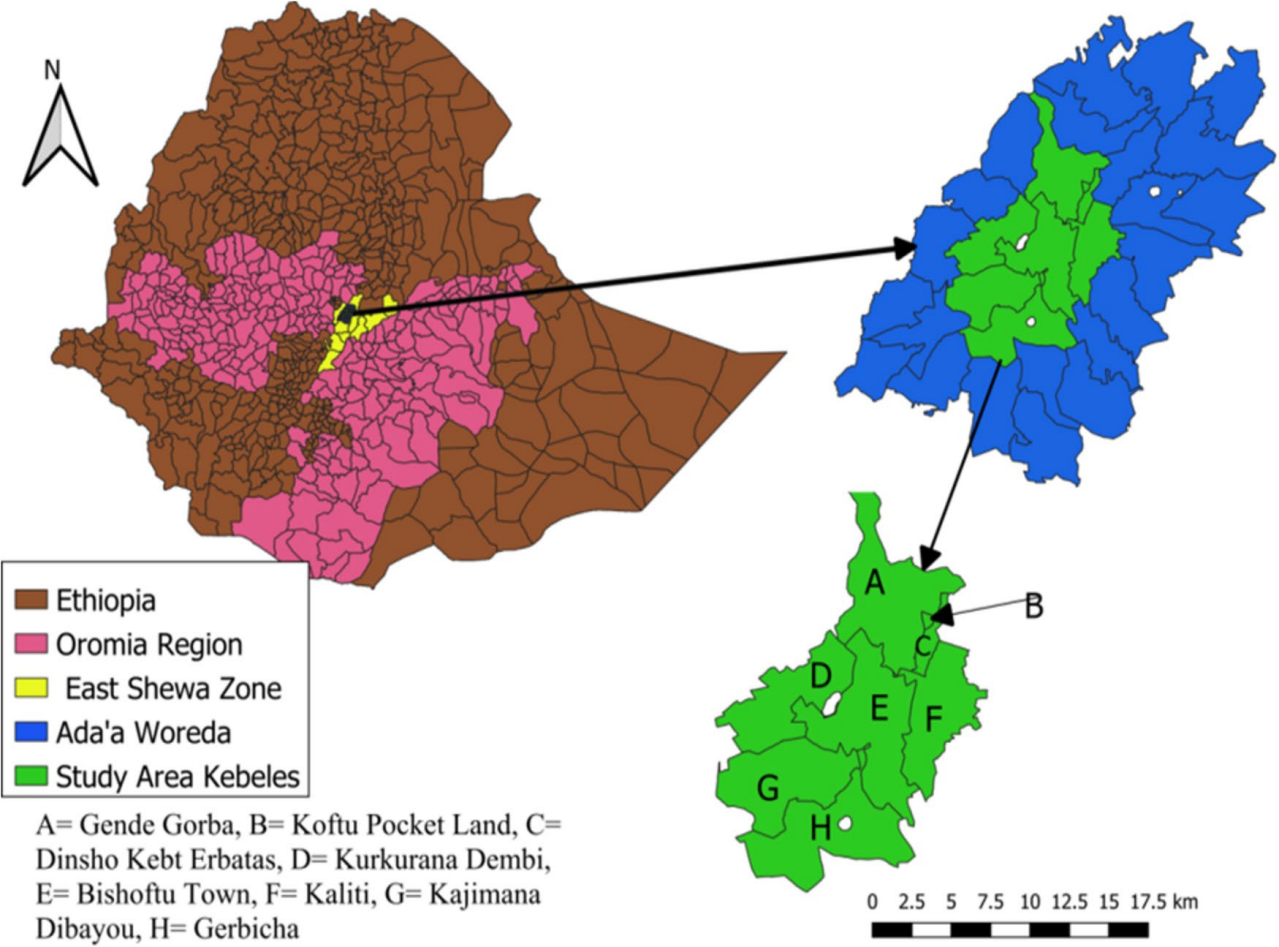
Map of Ethiopia depicting the location of the experimental site and the origins of the experimental animals, drawn using QGIS (https://qgis.org/en/site/)

This experimental study was conducted at the Veterinary Teaching Hospital of the College of Veterinary Medicine and Agriculture, Addis Ababa University, Bishoftu campus, which is 40 km Southeast of the capital city, Addis Ababa. The pneumonic sheep and goats used in this experiment were obtained from small holder farmers surrounding the hospital. The animals originated from Gende Gorba, Koftu Pocket land, Kaliti, Dinsho Kebt Erbatas, Kurkura Dembi, Kajimana Dibayou, Gerbicha and Bishoftu town (Fig. 1).

### Study animals and study design

The study was conducted on indigenous breeds of sheep and goats brought to the Veterinary Teaching Hospital of the College of Veterinary Medicine and Agriculture, Addis Ababa University, with clinical evidence of respiratory disease. Sheep and goats presented to the hospital were clinically examined for coughing, dyspnea, bilateral nasal discharge, and abnormal lung sounds. In addition, vital signs such as body temperature, heart rate and respiratory rate were recorded for each animal. Fifty-four sheep and goats were found to have these clinical signs and suspected of bacterial pneumonia and; were recruited for this study. The animals were randomly assigned to five experimental groups using the lottery system (G1: *n* = 12, G2: *n* = 11, G3: n = 11, G4: *n* = 9, G5: n = 11) and received OTTC 10% (Phenxyl, Phenix, Belgum, 10 mg/kg) IM for 3, 4, 5, 6, and 7 consecutive days. The animals were treated daily and followed up to record the vital signs and respiratory signs at the end of the treatment. Lung ultrasonography (L-USG) was performed before and at the end of therapy for each group. Nasal swabs were collected from each animal before and at the end of antibiotic administration for isolation and identification of *M. hemolytica* and *P. multocida*. Treated sheep and goats were returned daily to the owner’s farm until completing their therapy. The animal owners were instructed to care for their treated animals in isolated and well-ventilated pens with provision of palatable feed and potable water.

### Lung ultrasonography

**Fig. 2:**
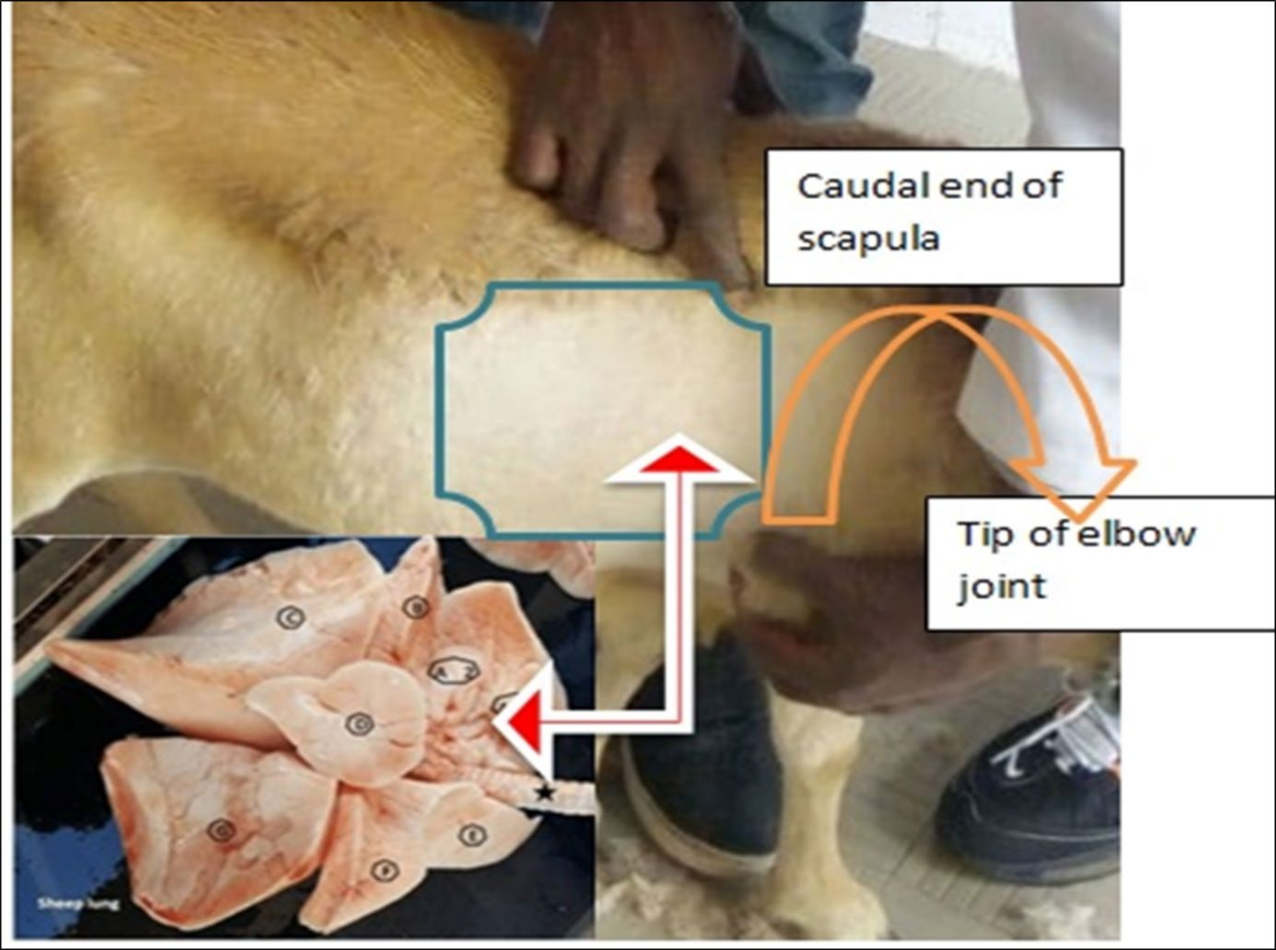
The anatomical area of the chest prepared for ultrasound examination

The progress of therapy was monitored using L-USG, Aloka SSD 500, Japan (https://www.medwrench.com/documents/view/1859/aloka-ssd-500-service-manual). The hair coat of the experimental animals was shaved on either side of the animals, cleaned and disinfected with alcohol and savlon while they were in a standing position. The area extending from the point of elbow to caudal tip of scapula dorsally and to ziphoid cartilage ventrally was demarcated. The specific anatomical site prepared for ultrasound examination is depicted in Fig. 2. The coupling gel was used over the shaved area, and the ultrasound sector abdominal probe with 7.5 mH was placed in the intercostal space and moved repeatedly down across the thoracic wall. The entire lung area was scanned and the lung images captured were frozen out from the USG screen, coded and characterized for any changes.

### Nasal swab sample collection

The nasal swab samples were collected aseptically after disinfection of the external part of the nostril with 70% alcohol. Sterile cotton swabs were moistened with casein digest tryptone soya broth (TSB, HIMEDIA, India), inserted into nostrils and gently rotated as described previously by Lobez et al., 2019 [12]. The swabs were placed into labeled sterile test tubes that contained 4–5 mL transport medium (TSB) and transported in an ice box to the Microbiology laboratory of the College of Veterinary Medicine and Agriculture. It was immediately incubated at 37 °C overnight to enrich the bacteria for bacteriological analysis.

### Bacterial culture and identification

Isolation and identification of *M. hemolytica* and *P. multocida* were performed following standard bacteriological techniques. Enriched nasal swabs (18 h) with TSB were directly streaked onto a blood agar base (Accumix Microexpress, India) supplemented with 7% defibrinated sheep blood and incubated at 37 °C for 48 hrs. The cultures were examined daily for evidence of bacterial growth. Colonies with typical phenotypic features consistent with *Pasteurella* species (Table 3) were further subcultivated on blood and MacConkey agar plates (HIMEDIA, India). Pure colonies grown on MacConkey (HIMEDIA, India) agar were transferred onto nutrient agar (HIMEDIA, India) for primary biochemical tests, such as motility using SIM medium (TITAN MEDIA, India) and catalase and oxidase tests. Secondary biochemical tests, including sugar fermentation tests such as glucose, sucrose, and lactose using TSI agar media (HIMEDIA, India) and production of indole (in SIM) were carried out according to the procedures illustrated by [13] for presumptive identification of the isolates. The presumptively identified pure colonies were preserved in 10% glycerol in TSB liquid medium for molecular confirmation.

### Molecular detection of *M. Hemolytica* and *P. multocida*

#### DNA extraction

For DNA extraction, pure cultures presumptively identified as *M. hemolytica* and *P. multocida* were transferred into 1.5 mL Eppendorf tubes. Genomic DNA was extracted using a BIONEER *AccuPrep* ^*@*^Genomic DNA Extraction Kit (k-3032) following the manufacturer’s protocol (Bioneer Corporation 8 11, Munpyeongseo-ro, Daedeok-gu, Daejeon 34,302, Republic of Korea). The DNA quality was checked by gel electrophoresis and stored at − 20 °C until required for further analysis.

#### PCR assay for detection of *M. Hemolytica*

Primers targeting virulence-associated genes coding for serotype-specific antigen *PHSSA* (*M. hemolytica* serotype-specific antigen) and *Rpt2* gene loci coding for methyl transferase of *M. hemolytica* (Table 1) were used for amplification as described previously [14]. The PCR was run with initial denaturation at 95 °C for 5 min, followed by 35 cycles of 95 °C for 1 min, annealing at 48 °C for 1 min, extension at 72 °C for 30 s and a final extension cycle at 72 °C for 7 min. One reaction tube with DNA template (reference *M. hemolytica* isolate from National Veterinary Institute (NVI)) and another tube with no DNA template were included as positive and negative controls, respectively.

**Table 1:** Primer pairs, amplification capacity and annealing T^o^ of targeted virulence-associated genes of *M. hemolytica* and *P. multocida* used in the study

Gene target	Primer sequence (5′----3′)	Size (bp)	Annealing T^o^ (°C)	Reference
*PHSSA*	Forward 5′- TTCACATCTTCATCCTC-3’	325	48	Kumar et al. (2015) [15]
	Revers 5′- TTTTCATCCTCTTCGTC-3’			
*Rpt2*	Forward 5′- GTTTGTAAGATATCCCATTT-3’	1022	48	
	Revers 5′- CGTTTTCCACTTGCGTGA-3’			
*KMT1T7*	Forward 5′-ATCCGCTATTTACCCAGTGG-3’	560	55	Shivachandra et al. (2006) [16]
*KMT1SP6*	Revers 5′- GCTGTAAACGAACTCGCCAC-3’			

#### PCR assay for detection of *P. multocida*

All PCR assay techniques applied for detecting *M. hemolytica* were similarly applied for *P. multocida* detection with the universal primers *KMT1T7* (F) and *KMT1SP6* (R), except for the cycling conditions. PCR thermocycling conditions were set with an initial denaturation at 95 °C for 5 min followed by denaturation at 95 °C for 45 sec, annealing at 55 °C for 1 min, extension at 72 °C for 1 min (35 cycles) and a final extension at 72 °C for 7 min. Negative (PCR mix without DNA) and positive (*P. multocida* isolate from NVI) controls were also included.

### Gel electrophoresis and analysis of the PCR products

The visualization of the amplified PCR product was undertaken by electrophoresis in 2% (w/v) agarose gel in 1x (TAE (Tris-acetate-EDTA)) buffer stained with 2 μL of Gel-red. Each PCR product (5 μL) was mixed with 6X loading buffer and loaded into separate wells of the preprepared gel, while 5 μL of 1 kb plus DNA molecular marker (DNA ladder) mixed with 2 μL loading buffer on aluminum plastic was loaded onto the first. Gel electrophoresis was run at 100 V for 90 min with an electrophoresis apparatus (EVOTEK#M12). The different band sizes of the PCR products were visualized under a UV transilluminator and photographed in a gel documentation system (BioDoc-It and VisiDoc-It Imaging Systems, TS software).

### Data management and analysis

These data collected on clinical parameters and vital signs were converted into scores, transferred to a Microsoft Excel spreadsheet, and checked for consistency. The scoring was conducted as follows: A) body temperature: normal (0) and above normal range (1); B) respiratory rate: normal (0), above normal range (1); C) heart rate: normal (0), above normal range (1); D) nasal discharge: no discharge (0), seropurulent (1), mucopurulent (2); E) coughing: no coughing (0), intermittent coughing (1), frequent coughing (2); F) L-USG lesion: no lung lesion (0), visible lung lesion (1); G) bacteriological findings; G1) *M. hemolytica*: negative (0), positive (1); and G2) *P. multocida*: negative (0), positive (1). Cumulative scores were computed for each animal. The data were then transferred to STATA software Version 17 for statistical analysis. A random effect-ordered logistic regression model was fitted to estimate the optimal therapeutic duration [17]. Data were also transferred to the R-program to generate a box-plot graph.

## Results

### Vital signs and clinical parameters of experimental animals

At baseline (pretreatment), the mean body temperature, heart rate and respiratory rate of the experimental animals were 40.57 ± 0.77^o^c, 98.77 ± 8.08 beats per minute, and 39.05 ± 3.97 breaths per minute, respectively. These parameters dropped 38.82 ± 0.31^o^c, 76.87 ± 3.26 beats per minute, and 20.04 ± 2.62 breaths per minute, respectively, after completion of the treatment. Before the treatment was administered, 29 (53.70%) of the animals had serous nasal discharge where 25 (46.29%) of them had muco-purulent nasal discharge, and 44 (81.48%) of them had frequent coughing. During the posttreatment clinical examination, none of the study animals (0.00%) showed nasal discharge. Intermittent coughing was observed in 22.22% of animals in G1, G2, and G3 but not in animals in G4 and G5. Table 2 presents the results of pre- and posttreatment vital signs and clinical parameters of the study animals.

**Table 2:** Pre- and post-treatment vital and clinical data summary of animals considered in this study

Vital and Clinical Sign	Pretreatment					Post treatment				
	G1 n = 12	G2 n = 11	G3 n = 11	G4 n = 9	G5 n = 11	G1 n = 12	G2 n = 11	G3 n = 11	G4 n = 9	G5 n = 11
**Bod temperature**	40.77 ± 0.89	40.53 ± 0.82	40.64 ± 0.68	40.6 ± 0.90	40.28 ± 0.62	39.16 ± 0.18	39.03 ± 0.19	38.72 ± 0.15	38.50 ± 0.17	38.57 ± 0.14
**Hearth rate**	97.66 ± 9.33	100.91 ± 6.95	99.09 ± 6.77	100.00 ± 11.91	96.18 ± 5.17	79.83 ± 2.44	77.09 ± 1.86	76.54 ± 3.61	75.11 ± 2.20	75.18 ± 3.57
**Respiratory rate**	38.83 ± 4.21	40.83 ± 4.52	39.00 ± 3.82	39.11 ± 5.01	39.45 ± 2.54	22.83 ± 2.17	22.18 ± 1.16	18.13 ± 1.22	18.33 ± 1.22	17.54 ± 1.12
**Nasal discharge (n, %)**										
No nasal discharge	0	0	0	0	0	12 (100)	11 (100)	11 (100)	9 (100)	11 (100)
Sero-purulent	5 (42)	5 (45)	6 (55)	5 (55)	8 (73)	0	0	0	0	0
Muco-purulent	7 (58)	6 (55)	5 (45)	4 (45)	3 (27)	0	0	0	0	0
**Coughing (n, %)**										
No coughing,	0	0	0	0	0	6 (50)	7 (64)	9 (18)	9 (100)	11 (100)
Intermittent coughing	1 (8)	2 (18)	3 (27)	3 (27)	1 (9)	6 (50)	4 (36)	2 (82)	0	0
Frequent coughing	11 (92)	9 (82)	8 (73)	6 (73)	10 (91)	0	0	0	0	0
**Pneumonia detected by L-USG (n, %)**										
No	10 (83)	8 (73)	8 (73)	6 (73)	8 (73)	10 (83)	9 (82)	10 (91)	9 (100)	10 (91)
Yes	2 (17)	3 (27)	3 (27)	3 (27)	3 (27)	2 (17)	2 (18)	1 (9)	0	1 (9)
**Bacteriological (n, %)**										
*P. hemolyticus*										
No	9 (75)	2 (18)	6 (55)	6 (73)	4 (36)	12 (100)	11 (100)	11 (100)	9 (100)	11 (100)
yes	3 (25)	9 (82)	5 (45)	3 (27)	7 (64)	0	0	0	0	0
*P.multocida*										
No	7 (58)	8 (73)	9 (82)	8 (89)	9 (82)	12 (100)	11 (100)	11 (100)	9 (100)	11 (100)
Yes	5 (42)	3 (27)	2 (18)	1 (11)	2 (18)	0	0	0	0	0

## Results of lung ultrasonography

**Fig. 3:**
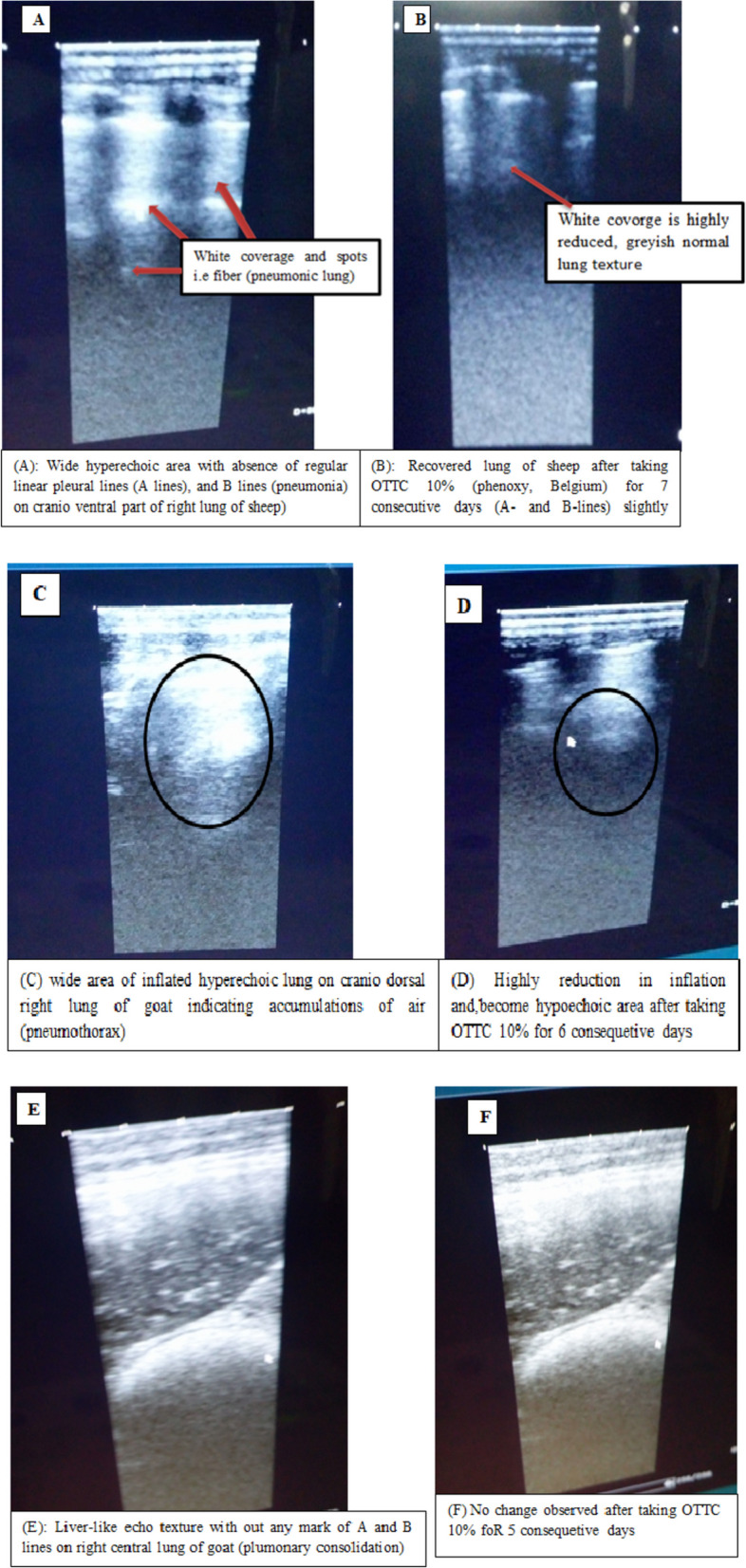
Lung ultrasonography images of a shirt at the VTH of CVMA-AAU. **a** Wide hyperechoic area with absence of regular linear pleural lines (A lines), and B lines (pneumonia) on cranio ventral part of right lung of sheep. **b** Recovered lung of sheep after taking OTTC 10% (phenoxy, Belgium) for 7 consecutive days (A- and B-lines) slightly. **c** wide area of inflated hyperechoic lung on cranio dorsal right lung of goat indicating accumulations of air (pneumothorax). **d** Highly reduction in inflation and, become hypoechoic area after taking OTTC 10% for 6 consequetive days. **e** Liver-like echo texture with out any mark of A and B lines on right central lung of goat (plumonary consolidation). **f** No change observed after taking OTTC 10% for 5 consequetive days

Ultrasound examination of the thorax revealed the occurrence of various lung lesions in 14 (25.93%) of the animals. The L-USG revealed that 8 (57.14%) animals with lung lesions had complete recovery. Experimental sheep and goats in G3, G4 and G5 had over 90.00% recovery rate (Table 2). The ultrasound detected pathological lesions lungs before and after treatment are shown in Fig. 3(A-E). There was a horizontally oriented mobile line in the recovered animals parallel to the pleural-lung interface. As shown in Fig. 3A, C, and E, there were no regular A and B lines. In contrast, in animals that were recovering from pneumonia after receiving treatment for 6 and 7 days, there were slight A and B lines appearing as the thickness of the lines decreased (Fig. 3B and D). There were anechoic areas of the lung and hyperechoic regions due to fibrin deposit and inflation due to accumulation of air (Fig. 3C). The consolidation and fibrin deposition decreased with the subsequent reduction in echoic areas after treatment (Fig. 3D). However, the hyperechoic consolidated area was still large in animals receiving treatment for less than 6 days (Fig. 3F). There was thinning of the pleural lines after treatment for 7 days (Fig. 3B).

### Presumptive identification of *Pasteurella* species

Out of the 54 nasal swabs collected and cultured during pretreatment, 27 (50.00%) yielded *M. hemolytica,* whereas 13 (24.07%) gave positive results for *P. multocida* (Table 3). However, none of the nasal swabs collected and cultured from the same animal after the completion of therapy yielded any positive results for *M. hemolytica* and *P. multocida*.

**Table 3:** Results of biochemical identification of *M. hemolytica* and *P. multocida*

Characteristics (tests)	Bacterial species	
	*M. hemolytica (n = 27)*	*P. multocida (n = 13)*
Hemolysis	+	–
Growth on MacConkey	+	–
Catalase	+	+
Oxidase	+	+
Indole	–	+
Lactose	+	–
Sucrose	+	+
Glucose	+	+

### Molecular identification of *M. Hemolytica* and *P. multocida*

**Fig. 4:**
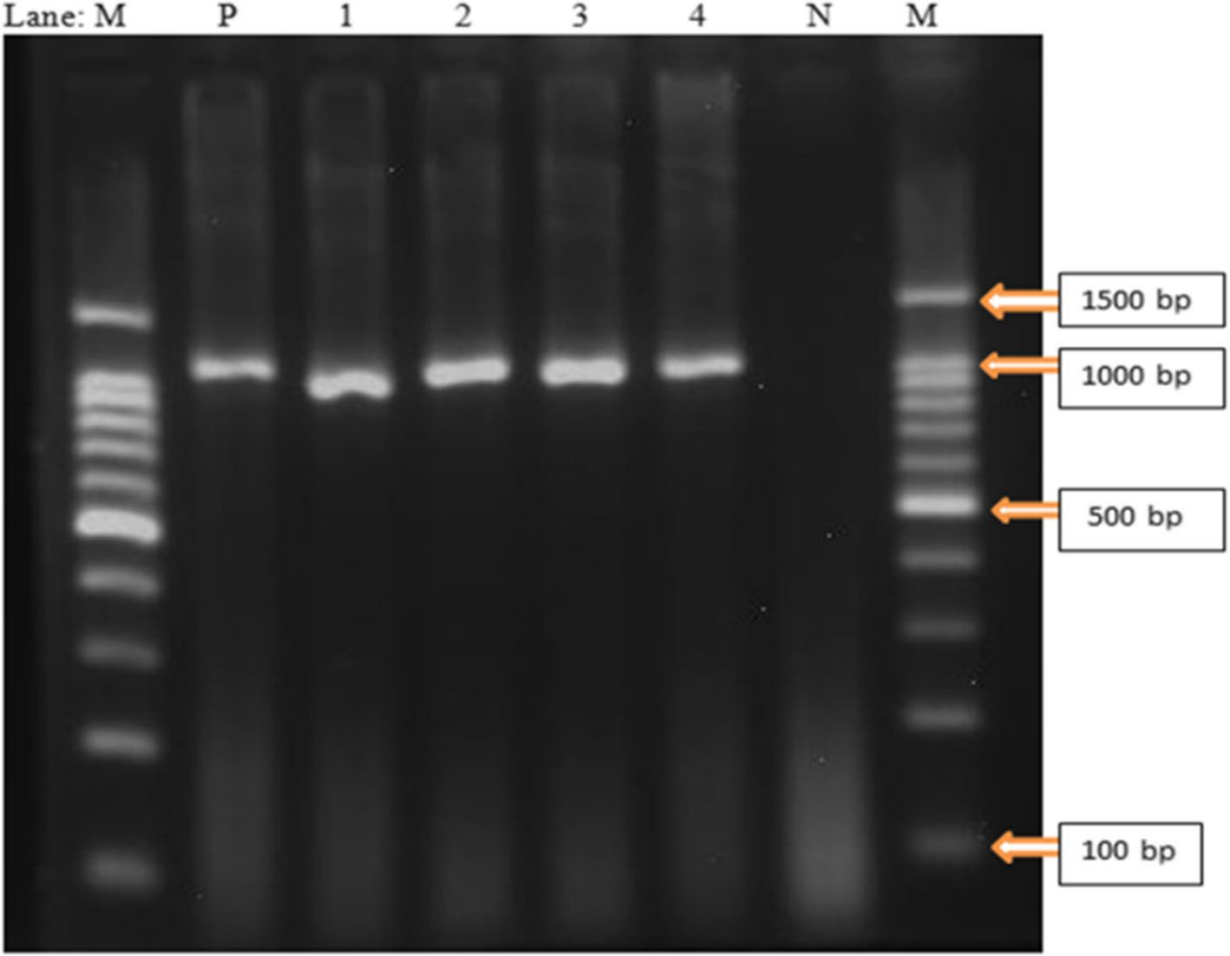
Agarose gel-electrophoresis showing PCR products (approximately 1022 bp) using primer pairs targeting virulence-associated genes (*Rpt2*) of *M. hemolytica*. Lanes: M = 1.5 kb plus DNA molecular marker (Smbio1.5 k DNA ladder), Lanes 1–4: *M. hemolytica isolates*. N: Negative control, P: Positive control from NVI (*M. hemolytica* type A) positive at approximately 1022 bp

Of the 27 isolates presumptively identified as *M. hemolytica,* 5 (18.52%) were confirmed by species-specific PCR. However, none of the 13 isolates identified as *P. multocida* based on the biochemical tests gave positive results in species-specific PCR. Both *PHSSA-* and *Rpt2-*targeted genes of *M. hemolytica* in a conventional PCR assay were used and 4 identified *M. hemolytica* were positive for the *Rpt2* gene (Fig. 4). Only 1 was positive for the *PHSSA* gene. Positive assays for both the *PHSSA* and *Rpt2* genes were not detected in this study.

### Optimized therapeutic duration and recovery

Respiratory disease remains the key challenge in small ruminant production. In Ethiopia, animals with signs and symptoms suggestive of respiratory diseases (pneumonia) are empirically treated with antibiotics for 3–5 days. The standard duration of antibiotic therapy for bacterial infections, including respiratory infections, is not based on evidence and is frequently unnecessarily short or long. In addition, the increasing prevalence of drug resistance as well as inadequate awareness of animal owners to bring their animals to veterinary clinics until the completion of therapy results in a low recovery rate and high mortality associated with respiratory diseases.

The results of this study revealed that in the pretreatment group, the median score of all variables considered in this study ranged from 6 to 7, whilst it ranged from 0 to 2 in the post-treatment group, demonstrating the resolution of most of the clinical and vital signs, bacterial identification and L-USG lesions. The data also indicate that among the posttreatment groups, the clinical and vital signs, the L-USG lesion the bacteriological infection resolve as the duration of therapy increases, with a median score of zero for animals in G3, G4, and G5. This corroborates the basic principle behind antibiotic treatment, as the clinical and pathological lesions start vanishing when diseased animals receive antibiotics for a minimum of 3 days (Table 3^3^).

**Fig. 5:**
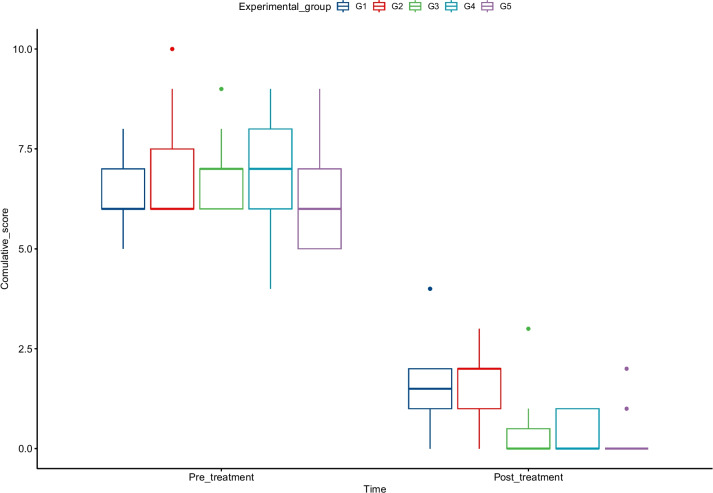
The median score plot of the cumulative outcome variables (clinical and vital signs, bacteriological, L-USG) in the study animal

The outcome variables (Table 4) were scored and entered into STATA software and fitted into a random effect-ordered logistic regression model to identify the optimal therapeutic duration by considering the cumulative scores of the outcome variables among the groups. Accordingly, the model indicated that the optimal time for the full recovery of an animal from respiratory disease was 7 days of treatment with OTTC. Considering all the variables in the model, the diseased sheep and goats that received therapy for 7 days (G5) showed the highest recovery rate compared to G1, and there was a statistically significant difference (coefficient (β) = − 2.296, P = 0.021) between G5 and G1. This suggests that symptomatic and clinical recovery from respiratory disease in sheep and goats was achieved with the administration of 10% OTTC for 7 consecutive days (full course) (Fig. 5).

**Table 4:** The medial scores of the outcome variables in the pretreatment and posttreatment shoats

Treatment groups	Number of animals	Median score		Coefficient	P- Value
		Pretreatment	Post-treatment		
Group 1	12	6	1.5	Ref	Ref
Group 2	11	6	2	0.564	0.505
Group 3	11	7	0	−0.959	0.266
Group 4	9	7	0	−1.416	0.139
Group 5	11	6	0	−2.137	0.021

## Discussion

In Ethiopia, respiratory diseases of sheep and goats are empirically treated with oxytetracycline (Phenxyl, Phenix, Belgium) or strepto-penicillin (Intercheme, Holland), usually for 3 days. The optimum duration of antimicrobial treatment is important for delaying the development of resistance and reducing the recurrence of clinical cases. In this study, we established the optimum duration of administration of oxytetracycline in sheep and goats affected with pneumonic pasteurellosis. This is crucial because the country is implementing control of PPR and major diseases of sheep and goats where respiratory disease complexes are also prioritized. In farm animals, antibiotics are used for the treatment of clinical diseases and for prophylactic purposes [18]. Veterinary and livestock authorities should take this into account while implementing prevention and control measures for respiratory diseases in sheep and goats.

At baseline (pretreatment), L-USG imaging found that 14 (25.9%) sheep and goats with clinical respiratory disease had images of various lung lesions with visual L-USG assessment. This finding is comparable to Tharwat et al. [19], who reported ultrasonic lesions in 13/55 (23.6%) of the diseased goats despite being latex agglutination test (LAT) positive for the presence of *Mycoplasma capricolum subspecies capripneumoniae (Mccp)* in all 55 goats before lung ultrasonography diagnosis in Saudi Arabia. The results in the current study were lower than the 31/31 (100%) ultrasound detection rate reported by Scot [20] in chronically infected sheep. The lower number of lung ultrasonic lesions obtained in this study from shoat with clinical respiratory disease could be due to either most animals brought to the clinic were at an early stage of disease development or most of them might be affected by upper respiratory diseases. Alternatively, as Scott [20] revealed, ultrasonography imaging can better diagnose and identify chronic lung lesions in sheep, including those that do not respond to antibiotic treatment. Ultrasonographic examination of the chest clearly defined superficial gross lesions which are consistent with a previous report [21]. Daily treatment with oxytetracycline for 7 days was successful in all sheep and goats diagnosed with pneumonia showing recession of the lesions although the impacts of operational or interpreting errors on this result are difficult to rule out. The L-USG imaging revealed that 8 (57.1%) had a total recovery rate, and sheep and goats in G3, G4, and G5 had an over 90% recovery rate. This shows that the pneumonia recovery rate increases as the treatment duration extends beyond 4 days.

Bacteriological culture and biochemical tests conducted on swabs collected before the treatment indicated that 27 (50.0%) isolates were positive for *M. hemolytica* and 13 (27.1%) for *P. multocida.* Molecular characterization using PCR indicated that 5 (12.5%) of the isolates were *M. hemolytica* and *none were P. multocida*, which is consistent with previous studies describing *M. hemolytica* as the most common agent isolated in cases of pneumonic pasteurellosis in shoat by Maru et al. [6] who reported 87.5% in Haramaya, Ethiopia, and Legesse et al. [22] who reported 92% in central Ethiopia. However, the *M. hemolytica* recovery rate in our study was lower than that reported by Legesse et al. [22] in central Ethiopia (34.21%); Alemneh et al. [23] in Fogera district (79.5%); Abera et al. [24] in Bedelle districts (46%); and Kaoud et al. [25] in Egypt (52%). The discrepancy in recovery rate among different study findings could be attributed to a variety of factors, such as geographical variation, sample size and season of sample collection, sampling site, and test sensitivity. The findings of this study revealed that neither *M. hemolytica* nor *P. multocida* was detected in post-OTTC 10%-treated animal nasal swab samples. The reason behind this might be due to the effect of this drug on the bacteria, which inhibits their growth [26] and hence the expression of the universal gene *P. multocida* (KMT1) and virulence-associated genes of *M. hemolytica* (PHSSA and Rpt2) targeted to be amplified by their respective primers in the current study. Hence, animals exposed to chemotherapy may not show the presence of *P. multocida* organisms in body fluid samples.

The results of clinical examination and recording of the vital signs in treated sheep and goats as well as ultrasonographic scanning of the lung revealed recovery rate as defined by return to normal parameters, and recession of the lung lesions increased as the duration of therapy increased from 3 days to 7 days. Despite the lack of previous research data on the impact of therapeutic duration, the possible explanations for this finding could be related to either the drug requiring repeated therapy to sufficiently interrupt the growth and proliferation of the sensitive bacteria in the infected animals or it could be due to involvement of several pathogens for disease development that takes longer time to respond or fail to respond due the nature of the involved organisms (gram-positive bacteria, virus, fungus or others) as oxytetracycline is more likely effective against gram-negative than other organisms [26]; resistance development to several or single organisms. As with all antibiotic classes, the antimicrobial activities of tetracyclines are subject to both class-specific and intrinsic antibiotic-resistance mechanisms [27]. Concurrent infections with many organisms and animal immunocompetence could possibly be factors in response delay since animal pathophysiology influences medication pharmacokinetics [28].

One of the key factors to be kept in mind is that treatments should be given for a sufficient length of time and that the appropriate drug is given in adequate doses by the most suitable route. In this study, we evaluated the optimal therapeutic duration for the effective recovery rate of shoats. Considering all vital and clinical signs, L-USG, and bacterial infection variables in the model, the diseased sheep and goats that received therapy for 7 days (G5) showed the highest recovery score compared to the other group, and there was a statistically significant difference (coefficient (β) = − 2.296, *P* = 0.021) in the variable score between G7 and G1. Although there have been no previous findings to substantiate our findings, the variation in the effect of the OTTC 10% in different therapy durations could be due to the drug’s residual effect on the pneumonic lung, which may be stronger in animals that take it for a long time. In human medicine, effective antibiotic treatment of nosocomial pneumonia has been noted to be for 7 days [29].

Our study, however, had several limitations: (1) the number of animals included in each group was not sufficient to generate statistically meaningful results (2) progressive or regressive clinical, vital, and other data were not continuously collected during the therapeutic duration; data were only collected at pre- and posttreatment periods, and (3) to avoid confounding factors, study animals were not kept under strict control conditions. In conclusion, our study suggests that symptomatic and clinical recovery from respiratory disease in sheep and goats was achieved with administration of 10% OTTC for 7 consecutive days (full course) and warrants a large-scale study to substantiate our findings.

### Supplementary Information

**Supplementary material 1.**

## Data Availability

All the data used for the generation of this manuscript is included. Raw data will be available from the corresponding author upon request.
