# Supplementary material for: Evaluation of the effect of therapeutic durations on small ruminant bacterial pneumonia
Source: BMC Vet Res. 2024 Feb 24;20:68. doi: 10.1186/s12917-024-03917-z (PMC10893678; doi:10.1186/s12917-024-03917-z)

Supplementary Figure

Two agarose gel photos, upper and lower

The lower gel photo is the one we used for this publication: Agarose gel electrophoresis showing PCR products (approximately 1022 bp) using primer pairs targeting virulence-associated genes (*Rpt2*) of *M. hemolytica*. From left to right Lanes: M = 1.5 kb plus DNA molecular marker (Smbio1.5k DNA ladder), Lane: 1 positive control from NVI (*M. hemolytica* type A), Lane 2–5: *M. hemolytica isolates*. Lane 6: Negative control


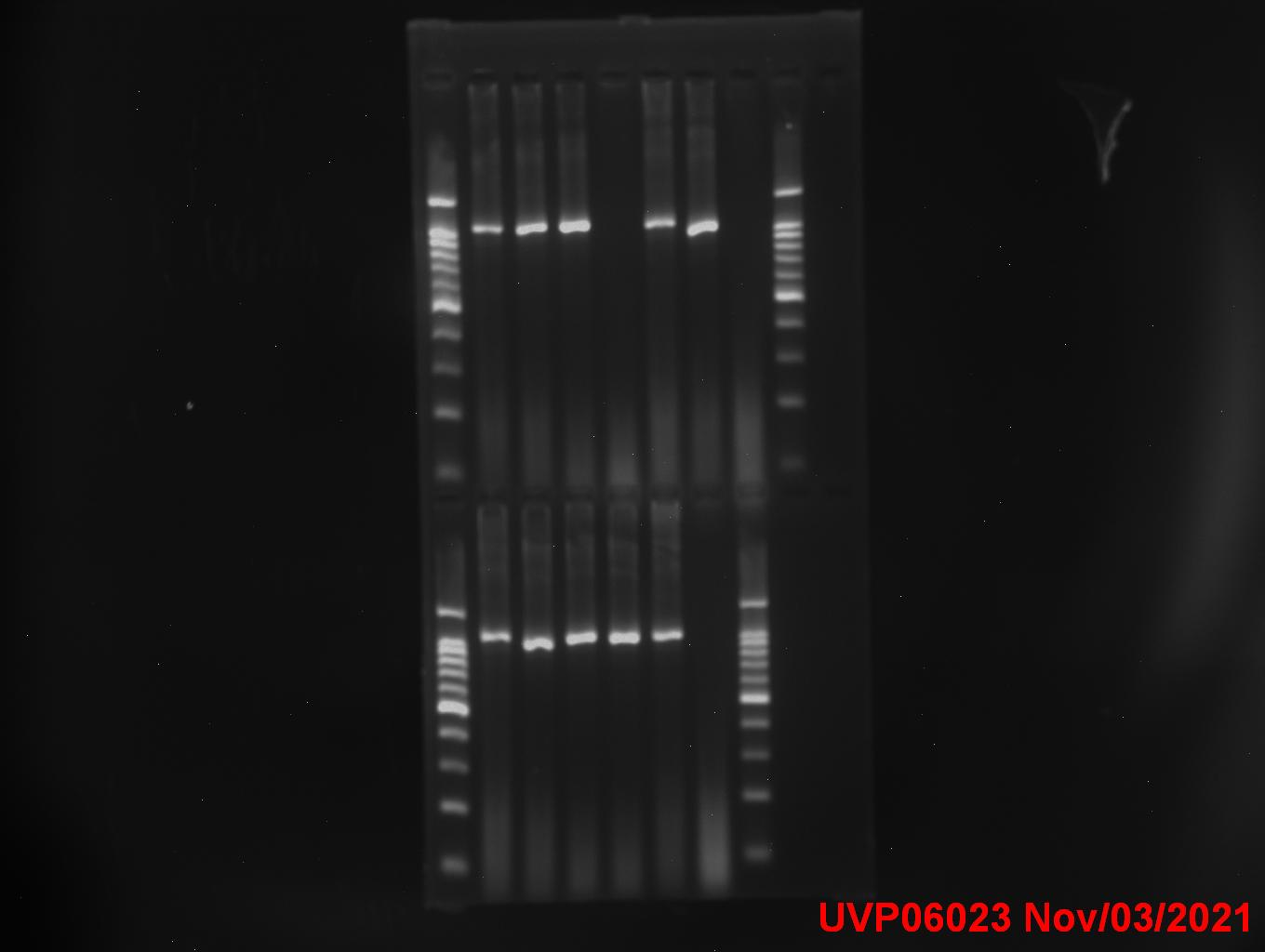

Supplement: Supplementary file 1 — Supplementary material 1. [file 12917_2024_3917_MOESM1_ESM.docx]
